# Accuracy of self-navigated free-breathing isotropic 3D whole heart inversion recovery magnetic resonance for detection of myocardial infarction

**DOI:** 10.1186/1532-429X-17-S1-Q121

**Published:** 2015-02-03

**Authors:** Tobias Rutz, Davide Piccini, Jerome Chaptinel, Simone Coppo, Gabriella Vincenti, Matthias Stuber, Juerg Schwitter

**Affiliations:** 1 Center for Biomedical Imaging (CIBM) & Center for Cardiovascular Magnetic Resonance Research, University of Lausanne, Lausanne, Switzerland; 2 Division of Cardiology, Cardiac MR Center, University Hospital Lausanne (CHUV), Lausanne, Switzerland; 3 Department of Radiology, University Hospital (CHUV) and University of Lausanne (UNIL), Lausanne, Switzerland

## Background

Cardiac magnetic resonance (CMR) allows detection of myocardial scar after myocardial infarction. Usually 2D image planes in short-axis and three long axis orientations are obtained. However to plan in patients with scar e.g. complex electrophysiological intervention for reentry arrhythmias, high-resolution 3D information of the scar is highly desirable. This study therefore evaluates the accuracy of self-navigated isotropic 3D-free-breathing CMR with inversion recovery (3D-SNIR) to detect myocardial scar tissue.

## Methods

Patients after myocardial infarction detected by late gadolinium enhancement on standard 2D inversion recovery sequences (2D LGE) underwent a CMR exam with 3D-SNIR on a 1.5T clinical CMR scanner (Aera, Siemens, Germany). Data acquisition was performed during the most quiescent systolic phase with a prototype segmented 3D radial trajectory with self-navigation after administration of 0.2mmol/kg of Gadobutrol. A non-selective IR pulse was added prior to each acquired k-space segment to the segmented, ECG-triggered, fat-saturated radial SSFP imaging sequence. Parameters: TR/TE 3.1/1.56ms, FOV (220mm)^3^, matrix 192^3^, isotropic voxel size (1.15mm)^3^, RF excitation angle 115°, and receiver bandwidth 898Hz/Px. TI (= 250-300ms) was assessed with a 2D radial scout scan prior to 3D-SNIR. A total of about 12'000 radial readouts were acquired for each 3D scan during free breathing with 100% respiratory efficiency. 3D LGE datasets were compared to standard 2D LGE for scar tissue detection with Osirix® software. Short axis 10mm slices were reconstructed from 3D LGE datasets by maximum intensity projection to yield a slice thickness of 10mm. Scar tissue was segmented on reconstructed slices on standard 2D and 3D-SNIR LGE and multiplied by slice thickness.

## Results

**Figure 1:**
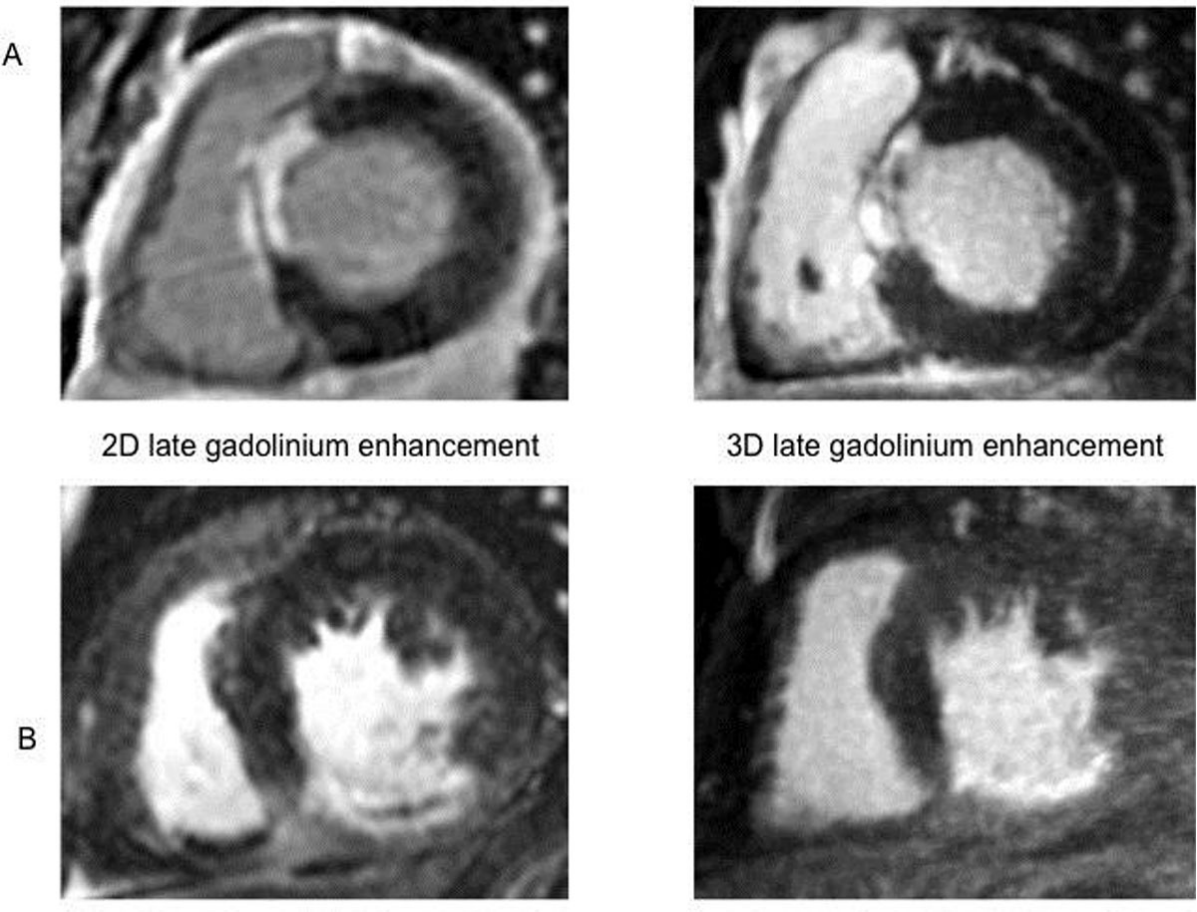
Examples for detection of myocardial scar in the septal (figure 1A) and inferior wall (figure 1B) by 2D (left) and 3D (right) late gadolinium enhancement in 2 patients.

**Figure 2:**
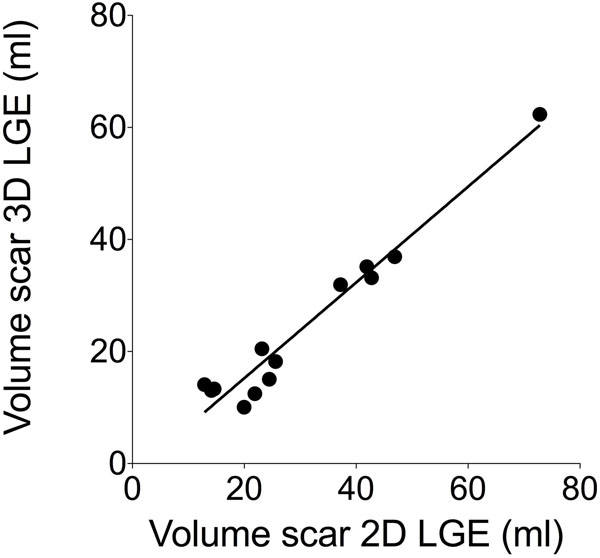
Correlation of volume of scar determined by 2D and 3D late gadolinium enhancement

Thirteen patients (5 females, age 58±10y) were included. Time between 2D LGE and 3D LGE was 59 ± 64 days. 3D-SNIR successfully corrected for respiratory motion in all acquisitions. All scars visualized by 2D LGE could be identified by 3D-SNIR (example see figure 1). Bland Altman-analyses and correlations showed a good agreement of quantification of scar volume obtained by 3D-SNIR compared to standard 2D LGE: -6.3±4.1ml, linear regression: r=0.977, p<0.001 (figure 2). 3D scar volume was 24.3±15ml vs. 2D 30.6±17ml, p<0.001. Intraobserver variability was 0.3±4.9ml, r=0.985, p<0.001; interobserver 1.5±9.9ml, r=0.74, p=0.014.

## Conclusions

Detection of myocardial scar by 3D-SNIR is feasible and shows a good agreement with standard 2D LGE. The mean difference of -6.3ml might be explained by the higher spatial resolution of the 3D sequence. Integration of a phase sensitive inversion recovery pulse warrants testing to further improve 3D quantification of scar.

## Funding

N/A.

